# Bactericidal Activity of Aqueous Acrylic Paint Dispersion for Wooden Substrates Based on TiO_2_ Nanoparticles Activated by Fluorescent Light

**DOI:** 10.3390/ma6083270

**Published:** 2013-08-02

**Authors:** Tommaso Zuccheri, Martino Colonna, Ilaria Stefanini, Cecilia Santini, Diana Di Gioia

**Affiliations:** 1 Department of Civil, Environmental and Materials Engineering, Alma Mater Studiorum-University of Bologna, via Terracini, 28, Bologna 40131, Italy; E-Mails: tommaso.zuccheri@unibo.it (T.Z.); martino.colonna@unibo.it (M.C.); 2 Department of Agricultural Sciences, Alma Mater Studiorum-University of Bologna, viale Fanin 42, Bologna 40136, Italy; E-Mails: ilaria.stefanini4@unibo.it (I.S.); cecilia.santini@unibo.it (C.S.)

**Keywords:** anatase aqueous dispersion, photocatalytic activity, titanium dioxide, fluorescent light, antimicrobial activity

## Abstract

The photocatalytic effect of TiO_2_ has great potential for the disinfection of surfaces. Most studies reported in the literature use UV activation of TiO_2_, while visible light has been used only in a few applications. In these studies, high concentrations of TiO_2_, which can compromise surface properties, have been used. In this work, we have developed an acrylic-water paint dispersion containing low TiO_2_ content (2 vol %) for the inactivation of microorganisms involved in hospital-acquired infections. The nanoparticles and the coating have been characterized using spectroscopic techniques and transmission electron microscopy, showing their homogenous dispersion in the acrylic urethane coating. A common fluorescent light source was used to activate the photocatalytic activity of TiO_2_. The paint dispersion showed antimicrobial activity against *Escherichia coli*, *Staphylococcus aureus* and *Pseudomonas aeruginosa*. The coating containing the TiO_2_ nanoparticles maintained good UV stability, strong adhesion to the substrate and high hardness. Therefore, the approach used is feasible for paint formulation aimed at disinfection of healthcare surfaces.

## 1. Introduction

The photocatalytic effect of titanium dioxide (TiO_2_) is a feasible and inexpensive technology, which has great potential in the disinfection of surfaces and matrixes [1]. TiO_2_ is able to generate free radicals when it is irradiated with a specific wavelength light [2]. It is well known that TiO_2_ is present in nature with three different crystalline structures: rutile, anatase and brookite. The different band gap energies of the three isomorphic forms of TiO_2_ explains the photocatalytic behavior responsible for the production of high oxidative chemical species that can interact in the degradation process of organic compounds and inactivation of viable cells, such as bacteria [3]. The most active form of TiO_2_ in the generation of free radicals is anatase, which releases radicals when exposed to ultraviolet (UV) light exceeding its band gap energy (3.2 eV corresponding to about 388 nm). In this way, the generated electron-hole pairs with redox effect on its surface do not recombine and can react with O_2_ and H_2_O in a continuous photocatalytic process, producing reactive oxygen species (ROS), such as superoxide radical anions (O_2_^•–^) and hydroxyl radicals (HO^•^) [2]. ROS production results in oxidative stresses towards microorganisms, leading to cell death [4,5,6,7]. For this reason, several studies report the application of TiO_2_ on surfaces as an antimicrobial agent under UV light source [8,9,10,11,12,13].

Dunlop *et al*. [14] and Evans and Sheel [15] reported that TiO_2_ thin films deposited onto stainless steel possessed high antibacterial activity against *Escherichia coli* and that the photocatalytic action was linearly proportional to the incident light intensity. These studies offer interesting application in the disinfection of drinking water. Vijay *et al*. [16] have reported that TiO_2_ nanocrystals synthesized under reactive plasma processing contain a higher anatase content with high crystallinity, which results in outstanding photocatalytic power for bacterial inactivation. Many researchers have investigated how to reduce significantly the bacterial survival rate by increasing the ROS formation [17,18,19,20,21,22]. These studies have contributed to the synthesis of new modified TiO_2_ nanoparticles, allowing, for example, for a better absorption of bacteria and chemical pollutants on particular substrates, such as textiles, and to the improvement of the antibacterial activity, thanks to the combination with specific metals, such as silver, gold or other photocatalytic semiconductor oxides. However, due to its large band gap, TiO_2_ needs the presence of UV light, such as sunlight, to be activated or a dedicated UV light source. Considering the damage to human cells induced by UV light, the use of the TiO_2_ photocatalytic process is confined to outdoor places or for specific uses [23].

In view of the possibility of applying the TiO_2_ photocatalytic activity of the anatase crystalline structure also to indoor applications with no damage for human cells, several studies have focused their attention on the possibility to shift the typical ultraviolet light TiO_2_ absorption towards the visible portion of the spectrum. Recently [1,24,25], several studies report the antimicrobial activity of TiO_2_ under fluorescent light (FL) against *E. coli*, *Staphylococcus aureus* and *Bacillus megaterium*. These studies show that the small UV light portion emitted by FL light is able to activate TiO_2_ to inhibit bacterial growth. In these works TiO_2_ nanoparticles have been deposited as a homogeneous film on a specific substrate. In another study [26], a close contact between TiO_2_ anatase crystals and bacteria was reached by depositing TiO_2_ powder on paper filters to which *E. coli* cells were also applied. Under illumination with FL light, a strong decrease of viable cells was evidenced. However, the inactivation rate did not increase upon increasing the TiO_2_ content. These results pointed out that once maximum cell-photocatalyst contact has been achieved, excess TiO_2_ does not enhance the anti-bacterial effect.

More recently, Caballero *et al*. [27] and Hochmannova and Vytrasova [28] have investigated the TiO_2_ photocatalytic activity of acrylic indoor paint formulation, which is raising great interest in the last few years for the disinfection of public places, including hospitals. However, in the former study, the photocatalytic agent was used at a very high concentration in the range of 15 vol %–80 vol %, and a significant bacterial inactivation of about 90% (investigated only against *E. coli*) was reached only after 48 h of irradiation. Furthermore, Hochmannova and Vytrasova [28] obtained an efficient antibacterial effect with the use of nano zinc oxide in an aqueous acrylic dispersion at the content of 5 vol %, whereas the bactericidal activity of TiO_2_ at the concentration of 7 vol % under common FL lamp was only 25% for the anatase dispersion. Moreover, a complete physical-chemical characterization of the photoactive materials used was not reported by the authors, neither was the study of the effect of such a high level of anatase on the mechanical properties and on the antimicrobial properties. Therefore, more studies are necessary to really understand the potentiality of TiO_2_ nanoparticles in the development of paint formulation with antimicrobial activity under FL. In particular, lower TiO_2_ content should be assayed, since the presence of large amounts of nanoparticles can have a detrimental effect on the mechanical and optical properties of the coated surfaces [29]. For this reason, in this study, paints with low TiO_2_ contents have been prepared in order to find out if it was possible to obtain a coating that can possess not only good antimicrobial activity, but also retain good UV stability, strong adhesion and good hardness.

In this work, the interest has been focused on the development of an anti-bacterial photocatalytic paint formulation, containing TiO_2_ anatase nanoparticles, for use in indoor applications, such as hospitals. Hospital-acquired infections are the sixth leading cause of death in the United States, and similar data are reported for Europe [30,31]. They are caused both by Gram-positive and Gram-negative bacteria [32]. In order to control bacteria attachment on surfaces and proliferation, it is important that the antimicrobial action occurs rapidly. The aim of this work is, therefore, the formulation of a solvent free aqueous dispersion containing a small amount of commercial TiO_2_ anatase nanocrystals (2 vol %) with an efficient and wide spectrum bactericidal effect activated by common indoor FL lights that can also retain good UV stability, good adhesion and hardness.

## 2. Results and Discussion

### 2.1. Preparation and Characterization of the AEROXIDE^®^ TiO_2_ P25 Coatings

The anatase nanocrystals have been completely characterized in order to evaluate the physical-chemical features able to promote the bactericidal activity of the prepared aqueous acrylic dispersion. Indeed, Aeroxide^®^ TiO_2_ P25 is a commercial product, and its physical-chemical characterization is already reported in the literature. However, conflicting data are reported in the literature on the crystalline content [33,34], and it is also reported that the composition of P25 can be inhomogeneous and changes depending on the position of sampling from the same package [35].

**Figure 1:**
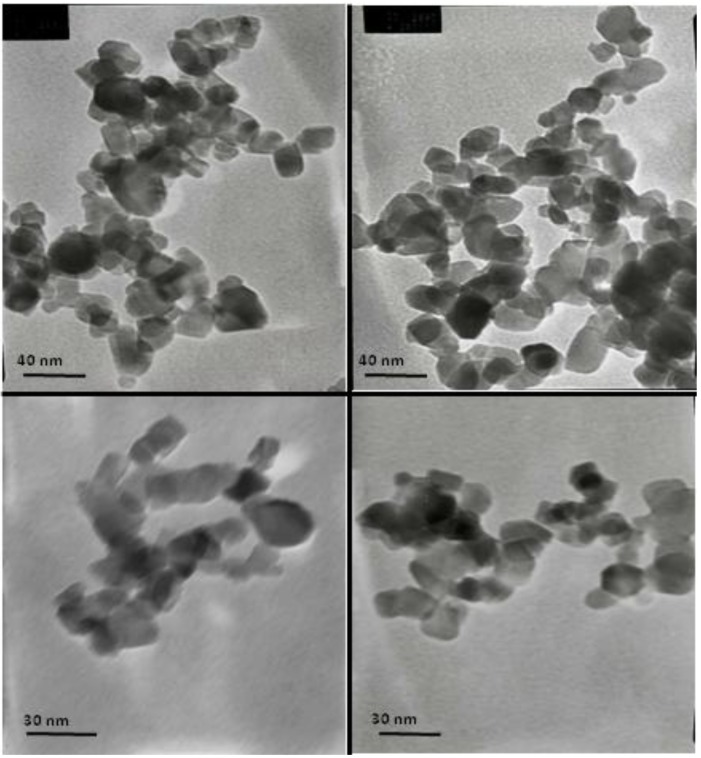
TEM images of TiO_2_ Aeroxide^®^ TiO_2_ P25.

**Figure 2:**
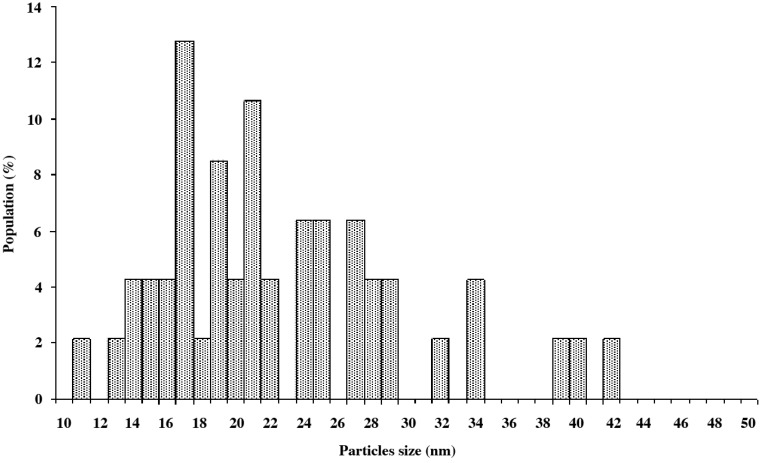
Aeroxide^®^ TiO_2_ P25 nanoparticles size distribution from TEM analysis.

It is well known that the photocatalytic properties are strictly linked to the TiO_2_ nanoparticles size and morphology [2], crystallite size, pore size [36,37] and amorphous content [38,39]. TEM images reported in Figure 1 show that the nanoparticles used in this study present an irregular faceted morphology and an average diameter size of about 22 nm, as evidenced in the histogram reported in Figure 2. The measured Brunauer-Emmett-Teller (BET) specific surface area of 43 m^2^ g^−1^ of TiO_2_ is in agreement with the analyzed crystal size and shape. The material used in the present study was found to be homogenous through all the samples used.

The elaboration of the XRD pattern (Table 1) showed that TiO_2_ was characterized by two main crystalline structures: 85% of anatase phase, and for the remaining, 15% of rutile.

**Table 1:** Physical properties of TiO_2_ Aeroxide^®^ P25 determined by XRD analysis.

Sample	XRD		TEM	Specific surface area (m^2^/g)
	Structure	Crystallite size (nm) ^a^	Average particles size (nm)	
Aeroxide^®^ TiO_2_ P25	Anatase:Rutile 85:15	24.2	22.6	43

^a^ Obtained by Scherrer equation.

**Figure 3:**
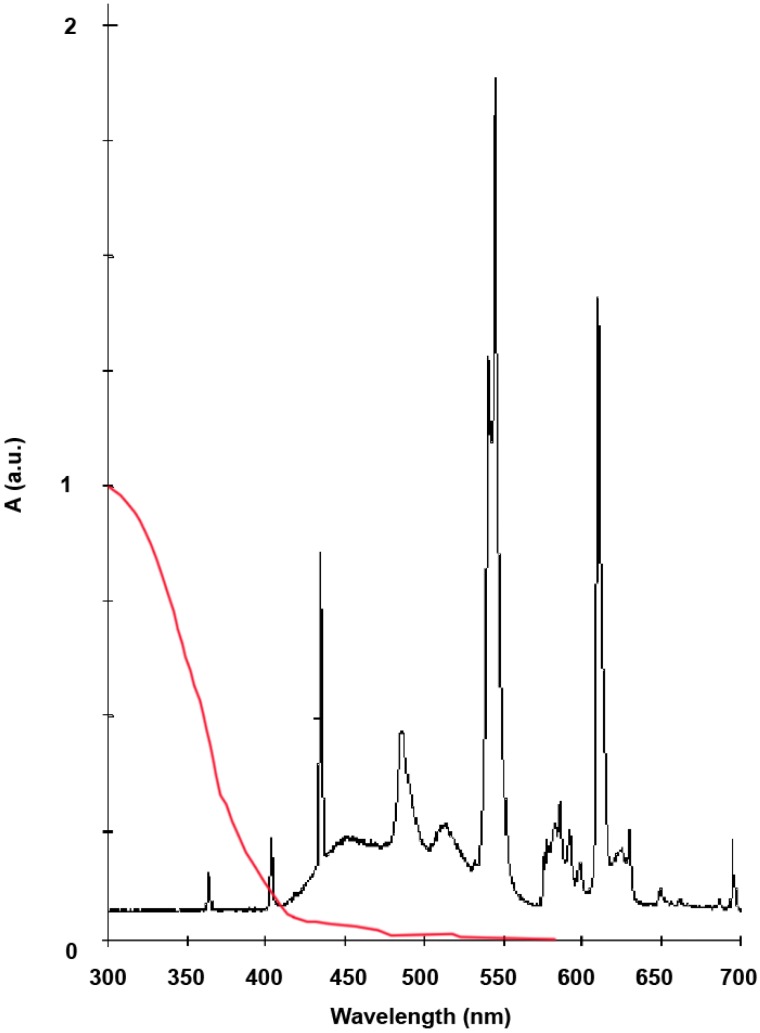
The solid-state diffusion-reflectance UV-Vis spectrum of Aeroxide^®^ TiO_2_ P25 (in red), and the UV-Vis emission spectrum of fluorescent light at 6500 K Daylight.

The absorption spectrum in the range 300–700 nm of the solid Aeroxide^®^ TiO_2_ P25 has been determined (Figure 3) showing a moderated intensity absorption in the visible region above 400 nm and a consistent absorption in the region comprised between 320 and 400 nm wavelength (UVA). For this reason, it is possible to have TiO_2_ photoactivation using a common FL for indoor environments, since the commercial FLs are characterized by a partial emission in the UVA spectrum region, as reported in the same figure (Figure 3).

**Figure 4:**
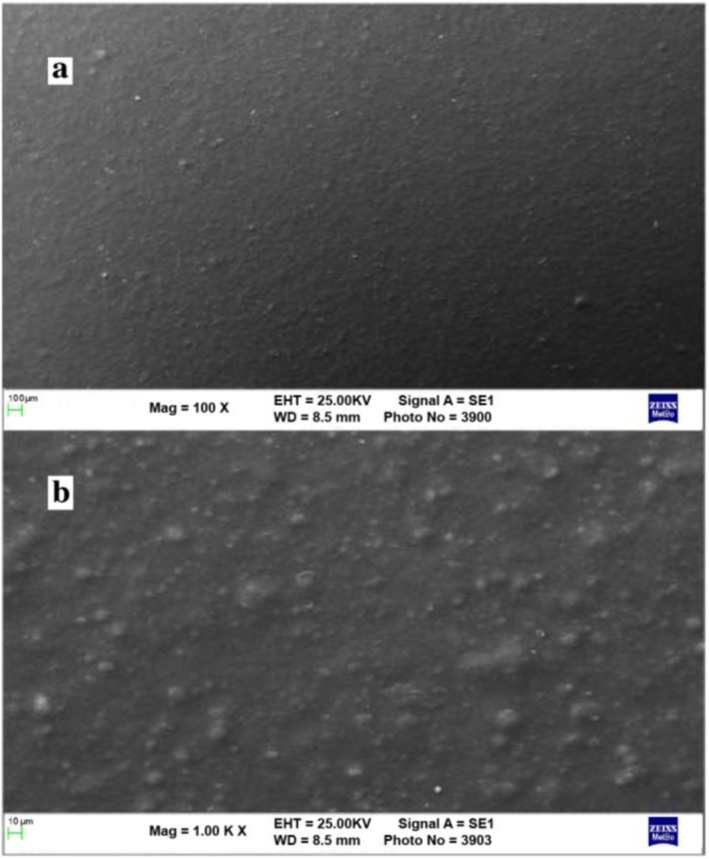
SEM images of the sprayed aqueous acrylic-urethane dispersion containing TiO_2_ nanocrystals at (**a**) 100×; and (**b**) 1000× magnification.

SEM analysis was also carried out in order to evaluate the morphology of the applied coating. Figure 4 shows that the acrylic urethane coating presents a micrometric substructure, due to the water-based paint formulation without any surface holes or significant roughness, irregularities and cracks.

**Figure 5:**
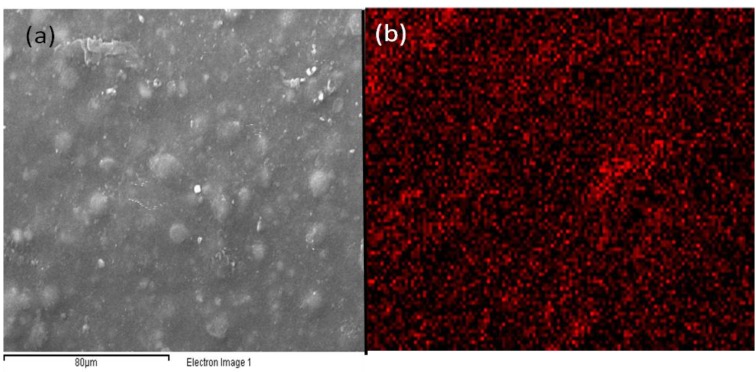
(**a**) SEM of aqueous TiO_2_ acrylic dispersion and (**b**) SEM-EDS of titanium electron emission of the same area.

The SEM-EDS analysis referred to titanium is shown in Figure 5b, while the SEM image of the same sample is reported for comparison in Figure 5a. Both pictures show that the TiO_2_ nanoparticles are uniformly dispersed through all the surface of the sample with no detectable agglomerations.

The coating was perfectly adherent to the support showing good physical properties, as confirmed by specific tests, such as cross-cut adhesion (according to norm UNI–ISO 2409/2007 [40], the coating has a result of zero, which means perfect adhesion); hardness (according to norm UNI 10782, with a result, H, which means high hardness). The tests show that the hardness and the adhesion of the coating, which are of fundamental importance in the final application of the paint, present good values after the addition of the anatase nanocrystals. The photoactive coating has strongly adhered to the lower substrate in a few minutes, thanks to the low minimum forming temperature and to the presence of the isocyanate catalyst, which determined a fast acrylic-urethane particle coalescence. The mechanical stability of the final coating film was the result of polymer chain inter-diffusion. In fact, the emulsion was a chemical cross-linked system in which the mechanical strength of the final coating is due to the reaction between the different components. Accelerated aging under UV light (QUV test according to norm UNI 9427/1989 [41] with a result of five, which means no degradation) show that the presence of the TiO_2_ anatase nanoparticles in this concentration has no detrimental effect on UV stability, which is one of the main issue when higher TiO_2_ contents are used [29].

### 2.2. Evaluation of the Antibacterial Activity

**Figure 6:**
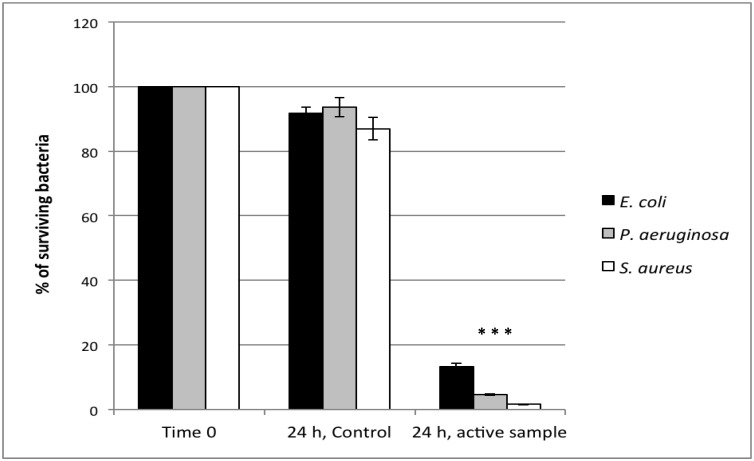
Percentage of surviving bacteria (*E. coli*, *P. aeruginosa* and *S. aureus*) on the control sample (without TiO_2_) and on the active sample (containing 2% w/w Aeroxide^®^ TiO_2_ P25) after 24 h of incubation at room temperature. The percentage of surviving bacteria was normalized to the inoculum (cells present at the beginning of the incubation). * *p* < 0.01 between T0 and T = 24 h.

In order to evaluate the bactericidal activity of the coating when activated by FL, bacterial strains (*i.e.*, *S. aureus*, *P. aeruginosa* and *E. coli*) were inoculated as a thin layer on the specimens containing 2 vol % Aeroxide^®^ TiO_2_ P25 and on the relative control (without Aeroxide^®^ TiO_2_ P25). The results of cell counts after 24 h of incubation at room temperature under FL are shown in Figure 6. Only a slight decrease of viable cells (statistically not significant) was evidenced in the control samples for all the three bacterial species assayed, whereas a significant decrease was observed in the Aeroxide^®^ TiO_2_ P25 containing samples. The percentage of viable cells after 24 h exposure was 23.2 for *E. coli*, 4.6 for *P. aeruginosa* and 1.7 for *S. aureus*. The three strains chosen for the test are all potential human pathogens and can cause trouble in hospitalized patients. *S. aureus* may occur as a commensal on skin, but it can infect other tissues when barriers are broken, thus causing severe diseases; it is reported to be the most common cause of nosocomial infections [42]. *P. aeruginosa* is an important bacterial pathogen, particularly as a cause of infections in hospitalized patients and immunocompromised hosts [43]. Surveillance of nosocomial *P. aeruginosa* infections has revealed trends of increasing antimicrobial resistance, leading to the conclusion that the most effective treatment is prevention. Moreover, mechanisms of virulence for this strain include the ability to colonize surfaces, forming biofilms. *E. coli* is one of the most frequent causes of many common bacterial infections. Thirty-five years ago, it was the most diffused Gram-negative bacterium associated with nosocomial infections, whereas in the last decade, it has been overcome by *P. aeruginosa* [31], although remaining a considerable threat. Therefore, the Aeroxide^®^ TiO_2_ P25 finely suspended in the acrylic-water paint dispersion at the concentration of 2 vol % possesses great photocatalytic bactericidal activity activated by FL against potential pathogens. The high inhibition percentages obtained after 24 h of incubation for all the three bacteria assayed make the approach used in this work feasible for the treatment of surfaces at sites where bacterial colonization has to be absolutely prevented, such as hospitals. The measured antimicrobial activity is higher with respect to that of other water-dispersed acrylic paint formulations with TiO_2_ nanocrystals reported in the literature. For example, Hochmannova *et al.* [28] report that the coatings containing TiO_2_ present a bacteria reduction of less than 30% at a concentration comprised of 2.5 to 5 vol % after 72 h of irradiation with visible light. In another study [27], total inactivation of *E. coli* occurred after 96 h when a very high TiO_s_ content (80 vol %) was used. It is difficult to make a hypothesis of the higher activity that we have obtained compared to previous studies, since the other works do not report any detailed information regarding the dispersion of the nanoparticles in the coating and on the type of substrate used for the coating. Indeed, the nature of the substrate can produce different results, since the surface area can be significantly different, and therefore, a change in antimicrobial activity can be expected.

## 3. Experimental Section

### 3.1. Chemicals

Aeroxide^®^ TiO_2_ P25 powder was provided by Evonik Degussa Gmbh (Hanau, Germany), and was used as photocatalyst in all experiments of this study.

The aliphatic urethane acrylic polyurethane copolymer emulsion (white translucent liquid, total solids 35 wt %, pH 8.2 (25 °C), viscosity Brookfield 37 (mPa·s; 25 °C), MFT 34 °C, density of emulsion 1.04 (20 °C; g cm^−3^)), containing 10 wt% of isocyanate catalyst, was obtained from Renner Italia S.p.A., Italy. The acrylic polymers are dispersed in water and are composed by microparticles stabilized in an aqueous medium with compounds improving the application on the substrate. Polymer microparticles create a binder, which makes it easier for the superficial film formation, and the specific surface active components are necessary to prevent polymer agglomeration within the dispersion. All products were not purified before use. All reagents for microbiological analyses were purchased from Sigma-Aldrich, Milan, Italy, except for the nutrient broth (NB), which was provided by Becton, Dickinson and Company (Franklin Lakes, NJ, USA).

### 3.2. Synthesis of the Acrylic Photocatalytic Paints

A 250 mL volume flask equipped with a magnetic stirrer was charged with 98.0 g of water-based acrylic urethane copolymer resin and maintained under stirring for 15 min. Two grams of Aeroxide^®^ TiO_2_ P25 nanocrystals were then added under vigorous stirring at room temperature until the dispersion was completely homogeneous. The amount of TiO_2_ nanocrystals added to the acrylic urethane copolymer resin was kept at low concentration (2 vol %) in order to obtain coatings with good mechanical and optical properties. The resin employed in this work is commonly used for indoor coating applications on various substrates. The coating was applied on the surface by a spray-coating technique. The coating thickness was 120 µm, as measured by scanning electron microscopy (SEM). The resin/Aeroxide^®^ TiO_2_ P25 mixture was deposited onto a wooden substrate by a spray-coating gun with a 2 mm nozzle and kept for 1 h at room temperature after coating application. The adhesion, hardness and accelerated aging under UV light have been measured using ISO standard tests. In particular, reference [40] with six blades and a cutting distance of 1 mm was used to measure the adhesion; the UNI 10782:1999 [44] pencil test was used for hardness measurements; the QUV test under [41] norm with a Xenon lamp was used to test the accelerated aging under UV light.

### 3.3. Morphological Characterization

Scanning electron microscopy (SEM) investigations have been carried out using a ZEISS (Oberkochen, Germany) EVO 50 EP electron microscope with an OXFORD INSTRUMENTS EDS INCA 350 microprobe (Abingdon, Oxfordshire, UK). The dried samples were glued by carbon tape on an aluminum stub and were gold coated.

Transmission electron microscopy (TEM) investigations were carried out using a Philips (Eindhoven, The Netherlands) CM 100 electron microscope at an accelerating voltage of 80 kV. The powdered samples were ultrasonically dispersed in distilled water and, then, deposited by dropcasting on conventional Formvar/Carbon 200 mesh copper microgrids.

### 3.4. XRD Analysis

X-ray diffraction powder patterns were collected using an Analytical X’Pert Pro (Eindhoven, The Netherlands) equipped with an X’Celerator detector powder diffractometer (Eindhoven, The Netherlands) using Cu K*α* radiation generated at 40 kV and 40 mA. The instrument was configured with a 1 divergence and 0.2 mm receiving slits. The samples were prepared using the front loading of standard aluminum sample holders, which are 1 mm deep, 20 mm high and 15 mm wide.

### 3.5. Specific Surface Area

Specific surface area measurements were undertaken using a Carlo Erba Sorpty 1750 (Milano, Italy) instrument by measuring N_2_ adsorption at 77 K and adopting the Brunauer-Emmett-Teller (BET) procedure.

### 3.6. Spectroscopic Characterization

Solid-state diffusion-reflectance UV-Vis spectra were measured in a quartz cell on a Perkin-Elmer Lambda 19 spectrophotometer (Cleveland, OH, USA) recorded at 25 °C in the 700–250 nm spectral region.

### 3.7. Evaluation of Photocatalytic Bactericidal Activity

A Gram-positive strain, *S. aureus* ATCC 6538P, and two Gram-negative strains, *E. coli* ATCC 11105 and *P. aeruginosa* M19, previously isolated from a hospitalized patient suffering from urinary infection (unpublished results) and available at the Department of Agricultural Sciences of the University of Bologna, were used as target microorganisms to evaluate the antimicrobial activity of the Aeroxide^®^ TiO_2_ P25 specimens. Each strain was grown aerobically in NB for 18 h at 37 °C. The specimens employed had a square shape (side length = 5.00 cm); three specimens containing 2 wt % Aeroxide^®^ TiO_2_ P25 and three control specimens with no antimicrobial agent were assayed for each strain. A transparent plastic cylinder with no bottom and top sides was applied to each specimen in order to border an area of 6.15 cm^2^. An overnight culture of each strain was obtained, the culture was centrifuged at 7000 × *g* for 10 min, the pellet was washed in sterile saline (8.5 g NaCl L^−1^) and resuspended in saline to obtain a cell suspension in the range of 2 × 10^7^–1 × 10^8^ CFU mL^−1^. An agar slurry solution was prepared dissolving 0.6 g of agar (Biolife Italia, Milan, Italy) in 100 mL saline; the solution was autoclaved at 121 °C for 30 min, cooled at about 40 °C and mixed in 1:1 proportion with the prepared cell suspension. The surface inside the cylinders was pre-wetted with sterile water, and 2.5 mL of the obtained agar slurry suspension were poured inside the cylinder. The agar slurry suspension was allowed to dry, a transparent glass was used to close the cylinder tops and the specimens were incubated at room temperature for 24 h under irradiation of an FL lamp (Megaman^®^ Electrical & Lighting LTD., Hong Kong, Model MU120i, 6500 K Daylight). The lamp emitted a wavelength of 370–400 nm, and the distance between the lamp and the samples was approximately 20 cm. The 24 h incubation time was chosen considering the protocol reported in the ASTM international standard E 2180-07 for the determination of antimicrobial activity in polymeric or hydrophobic material and according to results presented in the literature on the same topic [45,46]. The beginning and the end of incubation 0.1 mL of agar slurry was picked up; serial dilutions of this sample were performed and plated on Plate Count Agar (Biolife). The percentage of reduction of viable cells was calculated using the following equation:

% reduction = (a − b) × 100/a
(1)

where a is the number of viable cells (CFU mL^−1^) in the control specimen and b is the number of viable cells in the specimens containing TiO_2_. Statistical significance was calculated by comparing the two sampling times (0 and 24 h) with a *t*-test in each group, using the MEANS procedure (SAS).

## 4. Conclusions

This study demonstrates that nanoparticles of TiO_2_ in the anatase crystalline structure are suitable for the formulation of interior paints possessing anti-bacterial activity at low concentration (2 vol %). The commercial TiO_2_ nanoparticles containing 85% of anatase have been fully characterized and have proven to be uniformly dispersed in the acrylic urethane coating. The presence of the TiO_2_ nanoparticles has no detrimental effect on the mechanical and surface properties of the coating and, especially, on the UV stability. The UV light emitted by common FL is capable of ensuring the photocatalytic and antimicrobial effect against Gram-positive and Gram-negative bacteria frequently associated with hospital acquired infections, making the paint formulation particularly suitable for the disinfection of hospital surfaces.
